# Digital Health meets Hamburg integrated medical degree program iMED: concept and introduction of the new interdisciplinary 2^nd^ track Digital Health

**DOI:** 10.3205/zma001354

**Published:** 2020-11-16

**Authors:** René Werner, Maike Henningsen, Rüdiger Schmitz, Andreas H. Guse, Matthias Augustin, Tobias Gauer

**Affiliations:** 1 University Medical Center Hamburg-Eppendorf, Department of Computational Neuroscience, Hamburg, Germany; 2 University Medical Center Hamburg-Eppendorf, Center for Biomedical Artificial Intelligence (bAIome), Hamburg, Germany; 3 University Medical Center Hamburg-Eppendorf, Institute for Health Services Research in Dermatology and Nursing, Hamburg, Germany; 4 University Medical Center Hamburg-Eppendorf, Department for Interdisciplinary Endoscopy, Hamburg, Germany; 5 University Medical Center Hamburg-Eppendorf, Faculty of Medicine, Deanery, Hamburg, Germany; 6 University Medical Center Hamburg-Eppendorf, Department of Biochemistry and Molecular Cell Biology, Hamburg, Germany; 7 University Medical Center Hamburg-Eppendorf, Department of Radiotherapy and Radiation Oncology, Hamburg, Germany

**Keywords:** medical degree, digitalization, digital medicine, model degree program

## Abstract

Digitalization in medicine is transforming the everyday work and the environment of current and future physicians – and thereby brings new competencies required by the medical profession. The necessity for a curricular integration of related digital medicine and, in more general, digital health topics is mostly undisputed; however, few specific concepts and experience reports are available. Therefore, the present article reports on the aims, the implementation, and the initial experiences of the integration of the topic Digital Health as a longitudinal elective course (2^nd^ track) into the integrated medical degree program iMED in Hamburg.

## Zusammenfassung

Die Digitalisierung der Medizin verändert nachhaltig den Alltag und das Umfeld aktueller und zukünftiger Ärztinnen und Ärzte – und somit zugleich die im Arztberuf benötigten Kompetenzen. Die Notwendigkeit der curricularen Integration von Inhalten der Digitalen Medizin bzw. von Digital Health im Allgemeinen ist weitgehend unbestritten; konkrete Konzepte und Erfahrungsberichte sind jedoch bislang kaum zu finden. Im vorliegenden Bericht werden daher die Ziele, die Umsetzung sowie initiale Erfahrungen der Integration des Themenkomplexes Digital Health als longitudinaler Wahlpflichtbereich (2^nd^ Track) in den integrierten Modellstudiengang Medizin Hamburg (iMED) beschrieben.

## 1. Introduction

Health apps are already extensively being used [1], [2], [3] and the prerequisites and processes for their prescription have become a topic of current legislative debates [4]. But how can the benefits and quality of health apps be objectively recorded and assessed? Artificial intelligence (AI) and big data are widely anticipated to change, if not revolutionize, current medical practice [5]. AI systems have demonstrated to be on par with or even surpass human experts in specific tasks [6], [7]. But how do these systems function technically? What are their inner workings? How can, and should, they be integrated into daily clinical practice? What implications will this pose on data privacy and liability? These selected examples already illustrate that the digital transformation of the health care system, which goes far beyond mere digitization, will change the way in which current and future clinicians carry out their daily work. Accordingly, the questions arise as to which competencies clinicians will need in order to work effectively in these changing environments – and to actively shape the process themselves. And how should these competencies be integrated practically into the medical curricula [8]? As a first response, the Faculty of Medicine of the University of Hamburg developed a new interdisciplinary elective course *Digital Health,* which it introduced into the Hamburg integrated medical degree program (iMED) as of the winter semester 2018/2019. This report describes the underlying objectives, the specific implementation and structure of this course as well as the initial experiences.

### iMED elective courses: thematically structured longitudinal 2nd tracks

**Figure 1:**
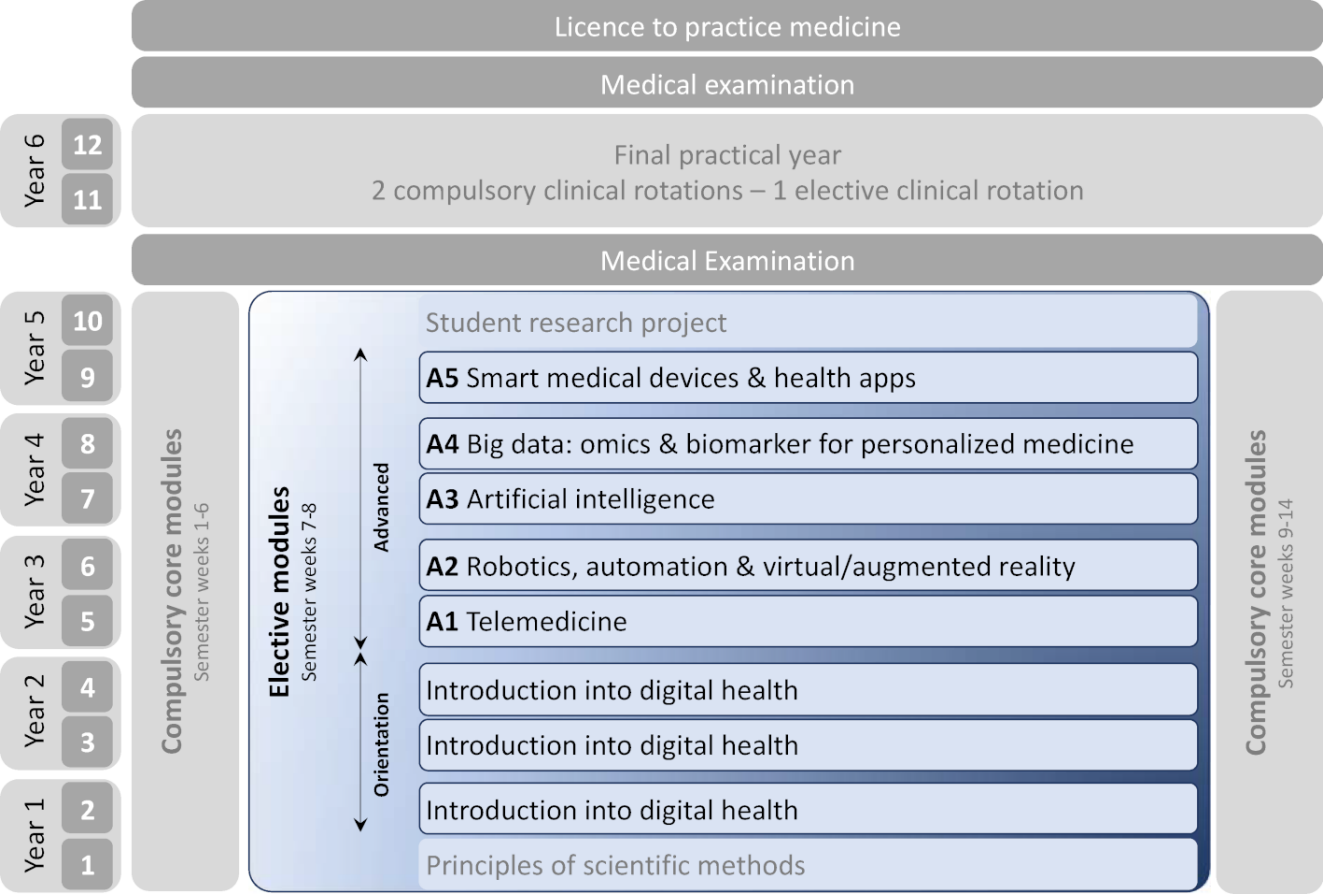
Structure of the 2^nd^ track Digital Health (blue) and its integration into the Hamburg iMED medical degree program (adapted from [12]). The orientation modules (semesters 2-4) do not build on each other. They present an overview of the topics of the advanced modules and thus form the basis of the selection of the field of specialization, which is taken by the students from the 5^th^ semester on.

The integrated medical degree program iMED consists of a modularly structured core curriculum and elective courses (the so-called 2^nd^ tracks). As shown in figure 1 (Fig. 1), the first nine iMED semesters are organized in a 6-2-6-week rhythm: two six-week compulsory modules with a two-week elective course module in between. The elective courses are longitudinally arranged. In the 1^st^ semester, all students attend a module on principles of scientific methods. Semesters 2-4 form an orientation phase: Each semester, students attend an orientation module for one of the different thematically structured 2^nd^ tracks (see [9] for further details). After the 4^th^ semester and based on their experience in the orientation modules, the students select one of the 2^nd^ tracks as a specialization area for the semesters 5-9. The 10^th^ semester is dedicated to a compulsory student research project, which the students prepare as an independent academic achievement. Thus, the subject-specific content to be offered by each 2^nd^ track comprises of a two-week orientation module (for students in semesters 2-4) and five two-week advanced modules (semesters 5-9). 

## 2. Requirements and planning of the new 2nd track Digital Health

The iMED 2^nd^ tracks are oriented toward the main research areas and interests of the University Medical Center Hamburg-Eppendorf (UKE) as well as the interests of the students; the necessity of a demand-driven extension and adaptation is evaluated regularly. The development of the 2^nd^ track Digital Health was initiated during the annual iMED faculty retreat in summer 2018 (focus topic: digitalization in medicine). To address the inherent interdisciplinarity and complexity of the topics digital medicine and digital health, the 2^nd^ track contents and goals were coordinated in open meetings and definition workshops for members of the Faculty of Medicine. In addition, active discussions and collaborations with the MIN faculty (Departments of Informatics and Physics) and the Faculty of Law of the University of Hamburg were sought. Furthermore, the medical students were offered to actively participate in the design and development of this new curriculum. 

### 2.1. Development of key 2nd track areas, comprehensive key questions, and a 2nd track matrix 

As the starting point for the 2^nd^ track development, the following overarching objectives were defined:

- The students will be introduced to the digital transformation process of the medical profession and its environment as well as the related opportunities and challenges of digitalization.
- The students will be enabled to critically discuss social and ethical questions and potential areas of conflict arising from the digitalization process.
- The students will ascribe to the interdisciplinary collaboration that these objectives require.

To select appropriate thematic foci to bring these generic objectives to life, the „Curriculum 4.0 – Medizin im digitalen Zeitalter“ (Curriculum 4.0 – Medicine in the digital age) introduced in Mainz in 2017 [8] served as a template. However, in contrast to the one-week elective course in Mainz, the structure of the iMED elective study program (see figure 1 (Fig. 1)) allows for the topics of digital medicine and digital health to be presented more broadly and in more depth. After intensive discussions, the following thematic foci were selected as the topics for the 2^nd^ track advanced modules:

- **Advanced module A1** (5^th^ semester students): telemedicine
- **Advanced module A2** (6^th^ semester): robotics, automation and virtual/augmented reality in medicine
- **Advanced module A3** (7^th^ semester): artificial intelligence
- **Advanced module A4 **(8^th^ semester): big data: omics and biomarkers for personalized medicine
- **Advanced module A5** (9^th^ semester): smart medical devices & health apps

The orientation modules (for students in semesters 2-4) were designed as an overview of the topics and contents of the 2^nd^ track. 

The concept design and implementation of the advanced modules of the 2^nd^ track was guided by five overarching questions:

- What are the main aspects and elements of digitalization in relation to the topics and applications covered by the respective module?
- What has been changed/improved by digitalization with regard to the module topics and patient treatment?
- What has been changed by digitalization with regard to the module topics for the medical profession and the required skills of the physicians?
- Which quality criteria were/are used to measure the benefit/value of digitalization with respect to the module topics?
- What open medical, technical, legal and ethical questions exist with regard to the module topics?

The elaboration of the five key questions for the five advanced modules and module-specific topics define the so-called 5x5 2^nd^ track matrix. This matrix serves as the thematic foundation of the 2^nd^ track that will be continuously adapted to developments in the health care sector.

#### 2.2. From theory to praxis: interdisciplinary and competency-driven distributed 2nd track organization 

The organizational effort for the development and implementation of a 2^nd^ track is immense. With up to four of the two-week modules of the 2^nd^ track, each with 30-40 teaching hours, being offered simultaneously, successful implementation requires collaboration and interdisciplinarity. To efficiently utilize the personnel resources of the UKE departments and institutes, the individual modules of the 2^nd^ track Digital Health are primarily organized independently and in a competence-driven manner. Thus, the A1 module is organized by the Department of Diagnostic and Interventional Radiology and Nuclear Medicine (contributing expertise in teleradiology) and the Institute for Health Services Research in Dermatology and Nursing (IVDP; e.g. teledermatology). The A2 module is coordinated by the Department of Radiotherapy and Radiation Oncology, the Department of Neurosurgery, and the Department of Urology (Martini Clinic). The A3 module is jointly organized by theoretically and practically oriented working groups (Department Institute of Computational Neuroscience, Department of Medical Systems Biology, the UKE Center for Biomedical Artificial Intelligence, and Department of Dermatology and Venereology). The Department of Cardiology and the Department of Pathology are responsible for the implementation of the A4 module, and the A5 module is coordinated by the IVDP. Course implementation and lecturing is further supported by the MIN faculty of the University of Hamburg and a student advisory board. The independent organization of the individual modules also incorporates the selection of the teaching formats. To further promote scientific application and principles, cross-module events (e.g. student research project fairs) are offered. Lastly, each module is to be concluded with a module examination. The appropriate form of examination in a 2^nd^ track Digital Health course is currently under discussion, but will be consistent for all advanced modules. For the orientation modules, the form of a multiple-choice exam was initially chosen; in the future – in line with the topic of the 2^nd^ track – electronic forms of examination will be introduced. 

## 3. Initial experiences and evaluation

The first orientation module was held in May 2019 with 13 students. The advanced modules will be introduced gradually, starting with the A1 module from the winter semester 2020/2021. Building on general iMED guiding principles and goals, special attention was given to the following aspects during the implementation of the orientation module:

- **Integration of theory and practice:** A key feature of iMED is the close integration of theoretical knowledge with practical skills, which is also reflected by the 2^nd^ track Digital Health. For example, the foundations and functional principles of virtual and augmented reality (VR, AR) are first introduced in a conventional seminar form and then directly concretized as part of a VR-based training course in anesthesia and emergency medicine offered at the UKE.
- **Critical attitude and problem awareness: **The digital transformation in medicine not only affects the medical environment and the doctor-patient relationship, but also the healthcare system as a whole. To illustrate this, an industry panel with three distinguished speakers both from academia and industry in the biomedical field was organized together with the research group Ethics in Information Technology at the Department of Informatics of the University of Hamburg. The invited panelists were encouraged to put on rose-tinted glasses and to present only the opportunities offered by the digitalization in their business sector, while the students were asked to identify the associated risks and potential ethical problems.
- **Development of communicative competencies:** In preparation for the industry panel, the student advisory board organized a so-called One Million Euro Challenge. The goal of the challenge was to directly apply the newly acquired knowledge of methods and applications (e.g. artificial intelligence) in small groups and to develop business models in the context of the digital transformation, i.e. to take the perspective of a digital health entrepreneur. In order to mimic a realistic “pitching scenario”, the developed business models were presented before and defended to the student advisory board that awarded the best idea and presentation. Each student group was supported by an experienced tutor. To structure and present the business models, concepts like SWOT analysis, the CO-STAR methodology [10] and the elevator pitch were tested together with the tutor.

“Exciting and practical perspectives!”, “Discussions were always very stimulating.” The students’ assessment in the final feedback round of our first run of the orientation module was consistently very positive. Furthermore, the official and student-questionnaire based evaluation of all 2^nd^ tracks, as conducted by the Faculty of Medicine, supported this feedback: The Digital Health orientation module received the highest scores in overall satisfaction (n=13 students, M=6.0, SD=0.0; 6=highest overall satisfaction, 0=lowest satisfaction) of all 16 2^nd^ track orientation modules offered in the semester. These initial experiences suggest that the presented concept is, in general, attractive. On the other hand, it should be noted that the orientation module offered 20 places for students but only the aforementioned 13 places were occupied. When asked, the students reported that many had suspected that this 2^nd^ track would contain “too much computer science stuff” – a fear that had been refuted. In addition, the 2^nd^ semester medical students considered the term “digital health” not clearly defined and sufficiently relevant when compared to classical medical elective course titles. This may seem surprising at first glance, but it confirms the observations that consumer level usage and familiarity of digital media does not necessarily lead to the acquisition of job-specific digital competencies [11] or a pronounced interest in them. 

## 4. Conclusions

The presented concept of integrating digital health as a longitudinal interdisciplinary 2^nd^ track into the Hamburg integrated medical degree program iMED enables related topics to be taught in greater breadth and depths than currently realized or intended in many other places. The concept thereby defines a model that can be transferred to and adopted by other university medical centers and medical faculties. In our view, there is, however, no alternative but to partner with external collaboration partners and to think beyond traditional faculty and university borders (see, e.g., the industry panel) to adequately address the complexity and dynamics of the field. The positive initial student feedback supports our ideas and concept, but represents only a first snapshot. The continued monitoring within the iMED quality assurance framework will show the extent to which digital health can be established permanently as a successful longitudinal elective course in iMED.

## Acknowledgements

The authors thank all colleagues and students that participated in the development, conception and realization of the 2^nd^ track (also and in particular those persons and UKE departments and clinics not mentioned in this article). We thank Dr. Leigh-Anne Dell-Brown for support with the English translation of this report. 

## Competing interests

The authors declare that they have no competing interests.
